# IoT-RECSM—Resource-Constrained Smart Service Migration Framework for IoT Edge Computing Environment

**DOI:** 10.3390/s20082294

**Published:** 2020-04-17

**Authors:** Zhongyi Zhai, Ke Xiang, Lingzhong Zhao, Bo Cheng, Junyan Qian, Jinsong Wu

**Affiliations:** 1 Guangxi Key Laboratory of Trusted Software, Guilin University of Electronic Technology, GuiLin 541004, China; zhaizhongyi@guet.edu.cn (Z.Z.); hisangke@gmail.com (K.X.); qjy2000@gmail.com (J.Q.); 2 State Key Laboratory of Networking and Switching Technology, Beijing University of Posts and Telecommunications, Beijing 100876, China; chengbo@bupt.edu.cn; 3 Department of Eletrical Engineering, Universidad de Chile, Santiago 1025000, Chile; wujs@ieee.org

**Keywords:** Internet of Things, edge computing, resource-constrained, smart service migration

## Abstract

The edge-based computing paradigm (ECP) becomes one of the most innovative modes of processing distributed Interneit of Things (IoT) sensor data. However, the edge nodes in ECP are usually resource-constrained. When more services are executed on an edge node, the resources required by these services may exceed the edge node’s, so as to fail to maintain the normal running of the edge node. In order to solve this problem, this paper proposes a resource-constrained smart service migration framework for edge computing environment in IoT (IoT-RECSM) and a dynamic edge service migration algorithm. Based on this algorithm, the framework can dynamically migrate services of resource-critical edge nodes to resource-rich nodes. In the framework, four abstract models are presented to quantificationally evaluate the resource usage of edge nodes and the resource consumption of edge service in real-time. Finally, an edge smart services migration prototype system is implemented to simulate the edge service migration in IoT environment. Based on the system, an IoT case including 10 edge nodes is simulated to evaluate the proposed approach. According to the experiment results, service migration among edge nodes not only maintains the stability of service execution on edge nodes, but also reduces the sensor data traffic between edge nodes and cloud center.

## 1. Introduction

The edge-based computing paradigm (ECP) is one of the most innovative modes of processing distributed Internet of Things (IoT) sensor data. In ECP, edge nodes form an intermediate layer extending from the cloud, and it brings computing, network, and service provision closer to the sensor-devices in IoT [1]. Therefore, ECP can overcome some defaults [2,3,4] in the cloud-based computing paradigm (CCP), such as high transmission delay and bandwidth bottleneck. In ECP, the edge layer is composed of many edge nodes, and it can provide supplementary capabilities for cloud centers. Presently, the ECP has been used into some smart services [5,6,7,8,9,10,11,12,13,14,15,16], because it can complete complex context-awareness and data analytics in real-time, and reduce the service latency and the whole network traffic [17].

In ECP, every edge node can be allowed to host a certain amount of services, which can be local services or offload services from cloud. The edge node determines the number of services according to the real-time state of node resources and service consumption. Nevertheless, in the running cycle of service, resource consumption is dynamically changed. In this way, the edge node may shutdown if the hosted services in it increase the required resources suddenly at some point and exceed the load capacity of the node. Due to the restriction of the size and cost, the devices of edge nodes always have limited resources and are hard to extend. In other words, the edge node is resource-constrained for hosting the services. Presently, most of the research [18,19,20] focuses on the service offloading from cloud to edge, without considering the resource constraint of edge devices.

To solve the above problem, this paper proposes a resource-constrained smart service migration framework for edge computing environment in IoT (IoT-RECSM) and a dynamic edge service migration algorithm. Based on this algorithm, the framework can dynamically migrate services of resource-critical edge nodes to resource-rich nodes by evaluating the edge node resource usage and edge service resource consumption. In the framework, a resource utilization model is presented to compute the synthetical occupancy rate of all kinds of resources of each edge node. The synthetical occupancy rate can be used to estimate whether the edge node needs to migrate some services to other nodes. A resource usage model is presented to compute the migrating probability value of each edge service. In addition, a migration service selection model is presented to determine which services would migrate to another node. An edge node selection model is presented to estimate whether the edge node can be selected as the destination of migration services by analyzing the edge node resource and migration delay. Finally, a prototype system is implemented to simulate the edge service migration in IoT environment. Based on the prototype system, an IoT case including 10 edge nodes is simulated to evaluate the effectiveness and performance of the IoT-RECSM. According to the experiment results, service migration among edge nodes not only maintains the stability of service execution on edge nodes, but also reduces the sensor data traffic between edge nodes and cloud center.

The remainder of this paper is organized as follows. Section 2 discusses the related work about smart service migration. Section 3 proposes the framework of IoT-RECSM, and describes the related models and algorithm of smart service migration. Section 4 designs a prototype system and gives a case. Finally, Section 5 concludes the paper.

## 2. Related Work

### 2.1. Service Migration Framework

Scheepers [18] introduced the features of some virtualization technology, for example, Xen hypervisor, Docker, Linux Containers (LXC), and presented the results of a comparison between the Xen and LXC virtualization technologies. Both hypervisor-based and container-based virtualization can provide portability, isolation and optimize the utilization of hardware resources. Le et al. [19] proposed a Cloud Service Selection with Criteria Interactions framework (CSSCI) that applies a fuzzy measure and Choquet integral to measure and aggregate non-linear relations between criteria. This Framework solved these critical issues of modeling the interactions between cloud service selection criteria, and designing indices to validate service selection methods. Kazzaz and Rychlý [20] proposed a RESTful-based framework for Mobile Web service migration and provisioning on both Android-based mobile devices and Java-based stationary devices in a P2P wireless network. This framework enables deploying, publishing, discovering, provisioning and migrating Web services to satisfy service providers’ and Web services’ preferences and improve Quality of Service (QoS) performance. Jeong et al. [21] proposed the Crystal framework that implementation of MapReduce on Crystal shows benefits of fog computing-fault-tolerant distributed processing over heterogeneous, unreliable, fog nodes while reducing overall latency. Wang et al. [22] proposed the ENORM framework. This framework can address the resource management problems of provisioning edge nodes for cloud applications, deploying workloads on provisioned edge nodes, and dynamic resource allocation on edge nodes. Happ and Wolisz [23] proposed a flexible IoT processing relocation framework. The framework dynamically and automatically selects a suitable execution location for processing tasks if those processing tasks should be computed on a Cloud server rather than the local gateway device. Ibrahiem et al. [24] proposed an architecture for transparent service continuity via double-tier migration (ARNAB) that is based on container migration. When ARNAB migrates a service (application), this architecture needs two tiers—the first tier migrates user connectivity, while the second tier migrates user containerized service. Puliafito et al. [25] provided a comprehensive summarization of both the existing virtualization techniques (e.g., Virtual Machine, Containers) and migration techniques (e.g., Cold migration, Pre-copy migration, Post-copy migration, VM migration), specifically examining their appropriateness for the network edge.

Most of the above frameworks focus on the service migration between cloud and edge nodes, such as ENORM, Crystal and relocation framework. The RESTful-based framework can transfer service between mobile devices and stationary devices to improve QoS, but this framework only considers the Migration Process Time, Battery Consumption and CPU Usage Consumption of devices. Moreover, container-based migration framework can effectively deal with the heterogeneity of different hosting environments for services. The IoT-RECSM will focus on the service migration among edge nodes to deal with the resource-constrained problem of edge devices. It also adopts the container-based technology to facilitate the service migration smoothly.

### 2.2. Service Migration Algorithm

Tziritas et al. [26] proposed an algorithm based on hyper-graph partitioning to solve the problem of simultaneously taking VM placement and replica placement decisions in tree-structured networks to reduce the overall network overhead incurred due to the communication dependencies between VMs and data. Bittencourt et al. [27] summarized two type migration strategies: non-live migration and live migration, and analyzed the scheduling problem in the edge computing environment, focusing on how user mobility can affect application performance and how three different scheduling policies, namely concurrent, FCFS, and delay priority, can be used to improve execution based on application characteristics. In addition, Zhao et al. [28] proposed the resource allocation scheme named Two-Dimension allocation and correlation placement Scheme (TDACP). It can establish a virtual machine placement strategy with high resource utilization efficiency and low time cost.

In the above algorithms, References [26,28] mainly focus on VM placement decisions for cloud service. Reference [27] presented a non-live migration strategy and a live migration strategy, in which the live strategy can effectively deal with the service termination, and non-live strategy can reduce the computational complexity of migration. By following the non-live strategy, the IoT-RECSM will design a distributed migration algorithm for the edge environment to dynamically manage the service migration.

## 3. IoT-RECSM Smart Service Migration Framework

**Figure 1:**
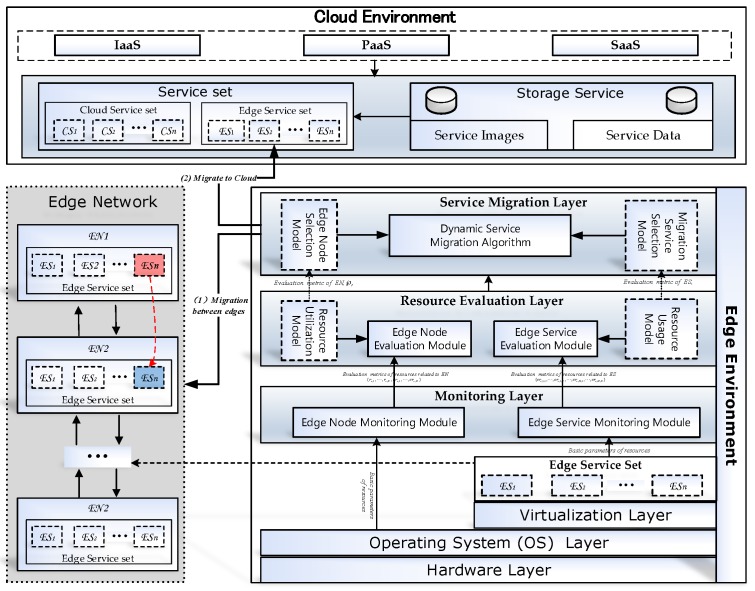
The framework of smart service migration.

In IoT-RECSM, the Cloud Environment takes charge of managing the services. According to the requirement of the application, the Cloud Environment may offload some services to edge nodes to improve the QoS. The Cloud Environment connects with the edge network of IoT. If the migrating service in edge network cannot choose a viable node, the Cloud Environment is also in charge of hosting the service. The Edge Network is an abstract decentralized topology of the IoT environment, in which each node $EN_{i}$ brings the computing, storage, and management for services, and follows the structure of Edge Environment as shown in the lower right-hand of Figure 1. The Edge Environment includes 6 layers: Hardware layer, Operating System (OS) layer, Virtualization layer, Monitoring layer, Resource Evaluation layer and Service Migration layer.

The hardware layer is used to abstractly represent all kinds of resources, for example, central processing units (CPUs), network adapter and memory, on the edge node (EN). Generally, the hardware of edge node is referred to as the lightweight devices, for example, mobile phones, network gateway. They have network communication, computation, storage, and intelligent auxiliary capabilities. Here, an abstract technology is also adopted in the hardware layer. So, the hardware layer can overcome the heterogeneity of resources on different edge nodes (ENs).

The Operating System (OS) layer is used to provide uniform hardware resource ports for the Virtualization layer and Monitoring layer of Edge Environments. OS layer not only can charge of monitoring the usage of all kinds of resources from the hardware layer, but also can manage the Virtualization layer for service deployment, service migration, and so forth.

The Virtualization layer is used to provide portability and optimize the utilization of hardware resources for edge services (ESs). This layer makes ESs easier to deploy, start, stop, and migrate.

The monitoring layer is used to collect resource consumption parameters from the OS layer and Virtualization layer. The monitoring layer includes two parts: the edge node monitoring module and the edge service monitoring module. The edge node monitoring module is used to collect the metrics of EN resources by getting the basic parameters from the OS layer. The edge service monitoring module takes charge of collecting the resource usage of each edge service by getting the basic parameters from the Virtualization layer.

The resource evaluation layer is designed to evaluate consumption situation of ENs and ESs by edge node evaluation module and edge service evaluation module. The edge node evaluation module is implemented based on the resource utilization model and can provide a synthetical evaluation metric of resource utilization for the edge node. The edge service evaluation module is implemented based on the resource usage model and can also provide a synthetical evaluation metric of resource usage for edge services.

The service migration layer is the core of the Edge Environment, which is used to migrate edge services. It includes three parts: Edge Node Selection Model, Migration Service Selection Model and Dynamic Service Migration Mechanism. The Edge Node Selection Model is presented to choose the displaced service in the resource-critical EN. The Migration Service Selection Model is presented to choose a well-resourced EN to adopt the displaced smart service. The Dynamic Service Migration Mechanism is designed to take charge of the service migration. A smart service migration algorithm is constructed for ENs. It can assist the EN to dynamically arrange the migration node when the EN is resource-critical. If none of EN is suited as the migration node, it migrates the service to Cloud. Table 1 lists some symbols that will be used in the next sections.

### 3.1. Resource Utilization Model for Edge Node Resource

In this section, the resource utilization model is defined to describe the evaluation mode of $EN^{\prime }$ s resources. Specifically, a synthetical occupancy rate is introduced as ${℘}_{v_{i}}$ as the evaluation metric. It can be computed by Equation (1).

(1) $${℘}_{v_{i}}=\sum_{k=1}^{r_{v_{i}}}\frac{exp(\frac{TCR_{v_{i},k}}{TR_{v_{i},k}})}{\sum_{k^{{}^{\prime }}=1}^{r_{v_{i}}}exp\left(\frac{TCR_{v_{i},k^{{}^{\prime }}}}{TR_{v_{i},k^{{}^{\prime }}}}\right)}\frac{TCR_{v_{i},k}}{TR_{v_{i},k}}$$

The ${℘}_{v_{i}}$ provides a normalization method for evaluating the utilization of the whole resources. In IoT-RECSM, the ${℘}_{v_{i}}$ can be computed by the edge node evaluation module, and the $TR_{v_{i},k}$ and $TCR_{v_{i},k}$ can be provided by the edge node monitoring module.

### 3.2. Resource Usage Model for Edge Service

The resource usage model is defined to describe the evaluation mode of edge services in the resource-critical node. It introduces a migrating probability value $S_{v_{i},j}$ as the evaluation metric. It can be computed by Equation (2).

The $l_{S_{v_{i},j}}$ also provides a quantitative method for evaluating the resource usage of edge service $S_{v_{i},j}$ in the $v_{i}$. In IoT-RECSM, the $l_{S_{v_{i},j}}$ can be computed by the edge service evaluation module, and the $SCR_{s_{v_{i}},j,k}$ and $TCR_{v_{i},k}$ can be provided by the edge node monitoring module.

(2) $$l_{S_{v_{i},j}}=\sum_{k=1}^{r_{v_{i}}}\frac{exp(\frac{SCR_{s_{v_{i}},j,k}}{TCR_{v_{i},k}})}{\sum_{k^{{}^{\prime }}=1}^{r_{v_{i}}}exp\left(\frac{SCR_{s_{v_{i}},j,k^{{}^{\prime }}}}{TCR_{v_{i},k^{{}^{\prime }}}}\right)}\frac{SCR_{s_{v_{i}},j,k}}{TCR_{v_{i},k}}$$

### 3.3. Migration Service Selection Model

If $v_{i}$ is a resource-critical node, it should select an edge service $S_{v_{i},j}$ to migrate to another node. In this section, a service selection model is constructed to handle such tasks. This model tries to migrate a service to release enough resources for edge nodes, and takes into account the service delay to ensure the service quality.

In the framework, the migrating probability value $l_{S_{v_{i},j}}$ of each service can be computed for the $v_{i}$. When an edge node is resource-critical, the resolution strategy is to choose the service that consumes the most resources in the node. The $l_{S_{v_{i},j}}$ provides a synthetical evaluation metric of resource consumption. According to above strategy, the selection model can be designed by choosing the maximum value in the set $l=\{l_{S_{v_{i},1}},l_{S_{v_{i},2}},...,l_{S_{v_{i},\left\|S_{v_{i}}\right\|}}\}$. It can be computed by Equation (3).

(3) $$ser=\underset{s}{\arg}\max_{1<s<\|S_{v_{i}}\|}l$$

### 3.4. Edge Node Selection Model

In this section, an edge node selection model is constructed to choose a proper node as the hosting place for the migration service. This model tries to find a resource-rich node as the hosting place with minimal migration delay.

To facilitate the construction of this model, edge nodes in the IoT environment are divided into two categories: the migrating node set and the hosting node set. It introduces a utilization threshold $\theta_{max}$ to divide these two sets of nodes. If ${℘}_{v_{i}}>\theta_{max}$, the edge node $v_{i}$ belongs to a migrating node, otherwise, $v_{i}$ is a hosting node. The set of hosting node and set of migrating node are represented as $E_{h}=\left\{v_{s},...,v_{h}\right\}$ and $E_{m}=\left\{v_{t},...,v_{m}\right\}$, respectively.

The model firstly uses Equation (4) to evaluate resource utilization of every node. And then, the nodes are labeled in two sets, that is $E_{h}$ or $E_{m}$.

(4) $${℘}_{v_{i}}^{{}^{\prime }}=\sum_{k=1}^{r_{v_{i}}}\frac{exp(\frac{TCR_{v_{i},k}+SCR_{s_{v_{i}},j,k}+\Delta_{k}}{TR_{v_{i},k}})}{\sum_{k^{{}^{\prime }}=1}^{r_{v_{i}}}exp\left(\frac{TCR_{v_{i},k^{{}^{\prime }}+SCR_{s_{v_{i}},j,k^{{}^{\prime }}}+\Delta_{k}^{{}^{\prime }}}}{TR_{v_{i},k^{{}^{\prime }}}}\right)}\frac{TCR_{v_{i},k}+SCR_{s_{v_{i}},j,k}+\Delta_{k}}{TR_{v_{i},k}}$$

The Equation (4) introduces a resource increment mechanism that evaluation reserves an extra space-$\Delta_{k}$ for the edge node. It can prevent the phenomenon of service jitter.

The $\Delta_{k}$ can be computed by Equation (5)

(5) $$\Delta_{k}=\sum_{k=1}^{r_{v_{i}}}\frac{exp(\frac{SCR_{s_{v_{i}},j,k}}{TCR_{v_{i},k}})}{\sum_{k^{{}^{\prime }}=1}^{r_{v_{i}}}exp\left(\frac{SCR_{s_{v_{i}},j,k^{{}^{\prime }}}}{TCR_{v_{i},k^{{}^{\prime }}}}\right)}SCR_{s_{v_{i}},j,k}$$

By using the Equation (4), a set of hosting nodes $E_{h}=\left\{v_{s},...,v_{h}\right\}$ can be constructed for migrating service $S_{v_{i},j}$. And then, the model should choose an optimal node from $E_{h}$ that can bring minimum delay for the $S_{v_{i},j}$. Here, the network maximum flow (NMF) [29] is introduced to evaluate the migration delay.

**Figure 2:**
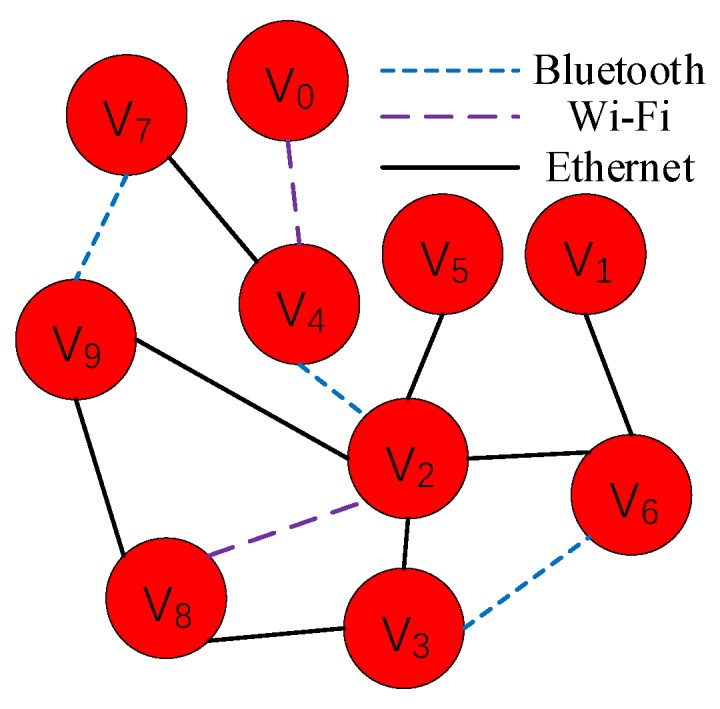
An edge network.

In the IoT edge environment, edge nodes are connected with each other in different communication modes, to form a network. Figure 2 shows an edge network, including 10 nodes and 3 communication modes. In the process of service migration, a non-live migration strategy is adopted to reduce the complexity of service management. That is, the whole of migrating service is transferred to node. Thus, the NMF method can precisely measure the migration delay based on the edge network model.

An edge network (ENN) can be abstracted as a pair of sets (V,E), where *V* represents the set of edge nodes and *E* represents the set of bandwidths on edge connections. The *V* is classified into two parts: the source nodes and the sink nodes. The source node *s*, that is, migrating node in above, needs to choose a service to migrate to another node. And the sink node *t*, that is, hosting node in above, can be as a destination of migrating services. Based on the edge network model, the network flow between two edge nodes can be defined as a function *f* that maps each bandwidth on edge connection *e* to a nonnegative real number *a*. This function can be represented as $f:E\to R^{+}$. The *f* satisfies the following two conditions: (1) for each edge connection *e*, the f(e) follows the relation $0\leq f\left(e\right)\leq c_{e}$, $c_{e}$ is the bandwidth on edge connection, (2) for each node v∈V, it follows the relation $\sum_{e\;into\;v}f\left(e\right)=\sum_{e\;out\;of\;v}f\left(e\right)$, the $\sum_{e\;into\;v}f\left(e\right)$ represents sum of communication traffic over all input connections of node *v*, the $\sum_{e\;out\;of\;v}f\left(e\right)$ represents sum of communication traffic over all output connections of node *v*. For a node v, the network flow $f=\sum_{e\;into\;v}f\left(e\right)$

NMF aims to compute the maximum transmission capacity from the migrating node to the hosting node. The Algorithm 1 shows the primary process of NMF. It first constructs an abstract model of edge network based on directed graph for the service migration. Then, it initializes f(e)=0, for every edge connection *e* in the network. Next, it searches all of paths from the source node *s* to the sink node *t*. For each path *p*, it gradually increases the up to the limitation $c_{e}$ of every connection *e*, to find out the threshold of communication capacity in the path *p*. Finally, the sum of thresholds in all paths is returned as the maximum flow for the service migration.
**Algorithm 1** Network Maximum Flow.**Require:**ENN,s,t **Ensure:***f*1: conversion the edge network into a directed edge network from *s* to *t*2: initialize flow *f* to 03: **while** there exists a path *p* between *s* and *t*
**do**4:    **if** all e∈p and $f\left(e\right)<c_{e}$
**then**5:      augment flow f(e) along *p*6:     **end if**7: **end while**8: **return**
*f*

For a migrating service $S_{v_{i},j}$, the migration delay $delay_{i,n}$ of every candidate node $v_{n}\in E_{h}$ can be predicted by the equation: $size(S_{v_{i},j})$/$NMF(ENN,v_{i},v_{n})$. The Node Selection Model will choose an edge node with minimal delay as the destination dest for $S_{v_{i},j}$, by Equation (6).

(6) $$dest=\underset{t}{\arg}\min_{t\neq i\;and\;1\leq t<\|E_{h}\|}\left\{delay_{i,n}\right\}$$

### 3.5. Dynamic Edge Service Migration Algorithm

In this section, a dynamic service migration algorithm is presented by combing the resource utilization model, resource usage model, service selection model and node selection model, as shown in Algorithm 2. This algorithm is provided for each edge node to complete the service migration automatically.
**Algorithm 2** Dynamic Edge Smart Service Migration.**Require:**$\theta_{max},v_{i},V,S_{v_{i}},TR_{v_{i},k},SCR,SCR_{S_{v_{i}},j,k}$**Ensure:**null1: ${℘}_{v_{i}}=\sum_{k=1}^{r_{v_{i}}}\frac{exp(TCR_{v_{i},k}/TR_{v_{i},k})}{\sum_{k^{{}^{\prime }}=1}^{r_{v_{i}}}exp\left(TCR_{v_{i},k^{{}^{\prime }}}/TR_{v_{i},k^{{}^{\prime }}}\right)}\frac{TCR_{v_{i},k}}{TR_{v_{i},k}}$2: **if**
$\theta_{max}<{℘}_{v_{i}}$
**then**3:  **while**$j<\|S_{v_{i}}\|$
**do**4:   $l_{S_{v_{i},j}}=\sum_{k=1}^{r_{v_{i}}}\frac{exp(SCR_{s_{v_{i}},j,k}/TCR_{v_{i},k})}{\sum_{k^{{}^{\prime }}=1}^{r_{v_{i}}}exp\left(SCR_{s_{v_{i}},j,k^{{}^{\prime }}}/TCR_{v_{i},k^{{}^{\prime }}}\right)}\frac{SCR_{s_{v_{i}},j,k}}{TCR_{v_{i},k}}$5:  **end while**6: $ser⇐get\;edge\;service\;which\;has\;the\;maximum\;value\;l_{S_{v_{i},j}}$7:  **while**
$v_{t}\in V\;and\;v_{t}\neq v_{i}$
**do**8:   $\Delta_{k}=\sum_{k=1}^{r_{v_{i}}}\frac{exp(SCR_{s_{v_{i}},j,k}/TCR_{v_{i},k})}{\sum_{k^{{}^{\prime }}=1}^{r_{v_{i}}}exp\left(SCR_{s_{v_{i}},j,k^{{}^{\prime }}}/TCR_{v_{i},k^{{}^{\prime }}}\right)}SCR_{s_{v_{i}},j,k}$9:   ${℘}_{v_{i}}^{{}^{\prime }}=\sum_{k=1}^{r_{v_{i}}}\frac{exp(TCR_{v_{i},k}+SCR_{s_{v_{i}},j,k}+\Delta_{k}/TR_{v_{i},k})}{\sum_{k^{{}^{\prime }}=1}^{r_{v_{i}}}exp\left(TCR_{v_{i},k^{{}^{\prime }}+SCR_{s_{v_{i}},j,k^{{}^{\prime }}}+\Delta_{k}^{{}^{\prime }}}/TR_{v_{i},k^{{}^{\prime }}}\right)}\frac{TCR_{v_{i},k}+SCR_{s_{v_{i}},j,k}+\Delta_{k}}{TR_{v_{i},k}}$10:   **if**
${℘}_{v_{i}}^{{}^{\prime }}<\theta_{max}$
**then**11:    $Add\;v_{i}\;to\;E_{h}\;set$12:   **end if**13:  **end while**14:  **while**
$v_{t}\in E_{h}$
**do**15:   $Get\;the\;maximum\;flow\;bandwidth_{i,t}\;between\;v_{i}\;and\;v_{t}$16:   $Computing\;the\;migration\;delay_{i}\;between\;v_{i}\;and\;v_{t}$17:  **end while**18:  $dest⇐get\;edge\;service\;which\;has\;the\;minimum\;delay_{i,t}$19:  **if**
dest==null
**then**20:   dest=cloud21:  **end if**22:  stopService(ser)23:  transfer(ser,dest)24: **end if**

This algorithm firstly computes the synthetical occupancy rate of device resources for the edge nodes, as shown in line 1. If $\theta_{max}<{℘}_{v_{i}}$, the edge node $v_{i}$ is resource-critical. It should choose a migrating service based on the service selection model (lines 3–6). And then, it gets the hosting node based on the node selection model (lines 7–13).

After getting the hosting node, this algorithm can compute the migration delay between $v_{i}$ and other nodes $v_{t}\in E_{h}$ by edge node maximum flow method (lines 14–17). Then, the algorithm is based on the edge node selection mode to get the migration destination dest. If none of nodes can host the service, it will be migrated to the cloud center (lines 18–21). Otherwise, it stops the running of service, and then transfers it to the hosting node, as shown in lines 22–23. Note that, the value of $\theta_{max}$ will influence the time of migration smart service and last effect the stability of edge node flow network.

## 4. The Prototype System and Case Study

This section firstly introduces the implementation the prototype system. And then, based on the prototype system, an IoT case including 10 edge nodes is simulated to evaluate the effectiveness and performance of the IoT-RECSM.

### 4.1. The Class Graph of Prototype System

**Figure 3:**
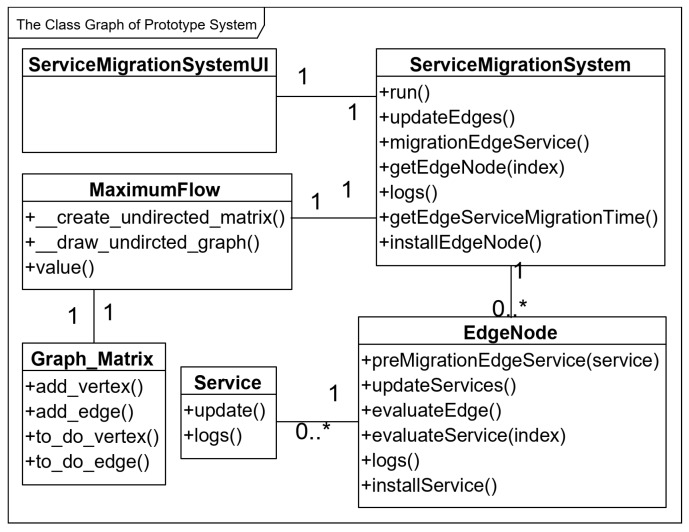
The class graph of prototype system.

The prototype system is developed according to the class graph as shown in Figure 3. The prototype system main includes 6 classes: *ServiceMigrationSystem* class, *EdgeNode* class, *Service* class, *ServiceMigrationSystemUI* class, *MaximumFlow* class and *Graph_Matrix* class. And the quantity relationship among the classes is shown in Figure 3. For example, The *ServiceMigrationSystem* class has a one-to-many relationship with *EdgeNode* class, and the *ServiceMigrationSystem* class also has a one-to-one relationship with *ServiceMigrationSystemUI* class.

The EdgeService class is an abstract representation of all the services on edge nodes. Service class uses the update() function to update the resource demand of service. The logs() function is used to output the logs that mainly include the number of various resources of each service.

In EdgeNode class, edge services are created by the installService() function. The edge node selection model is implemented by the preMigrationEdgeService() function to pre-migrate service from resource-critical edge nodes to resource-rich nodes and find out an optimal edge node as the destination for service migration. The resource usage model is implemented by the evaluateService() function. The resource utilization model is implemented by the evaluateEdge() function. The function updateServices() is used to update all services by calling the update() function of edge service.

The method of network maximum flow is implemented in MaximumFlow class, which can get the value of Maximum flow between two edge nodes. In MaximumFlow class, the _create_undirected_matrix() function is used to create adjacency matrices for edge nodes, respectively. The _draw_undirected_graph() function is used to generate a topology of edge nodes. Finally, the value() function returns the maximum flow value between two edge nodes.

The Graph_Matrix class is used to dynamically construct the network topology of edge nodes. In Graph_Matrix class, the add_vertex() function adds an edge node to the edge node network. The add_edge() function adds an edge to the network of edge nodes. The to_do_vertex() function returns all edge nodes in the edge node network. The to_do_edge () function returns all edges of the edge node network.

The ServiceMigrationSystem class is designed to create an instance of the prototype system. In ServiceMigrationSystem class, the installEdgeNode() function is used to instantiate all edge nodes. The updateEdges() function is used to call the updateServices() function of all edge nodes to update all edge nodes. The function migrationEdgeService() is used to migrate service from resource-critical edge nodes to resource-rich nodes. The run() function is used to start the simulation experiment, which will call the function updateEdges() to update all edge nodes.

The ServiceMigrationSystemUI is used to create a user interface (UI) of the prototype system. The next sub-section will detail introduce the UI of the prototype system.

In addition, the prototype system is developed with Qt Creator 4.10.1 (Qt 5.13.1) and Python 3.7.4, and the code is available online [30].

### 4.2. The Configuration of Prototype System

**Figure 4:**
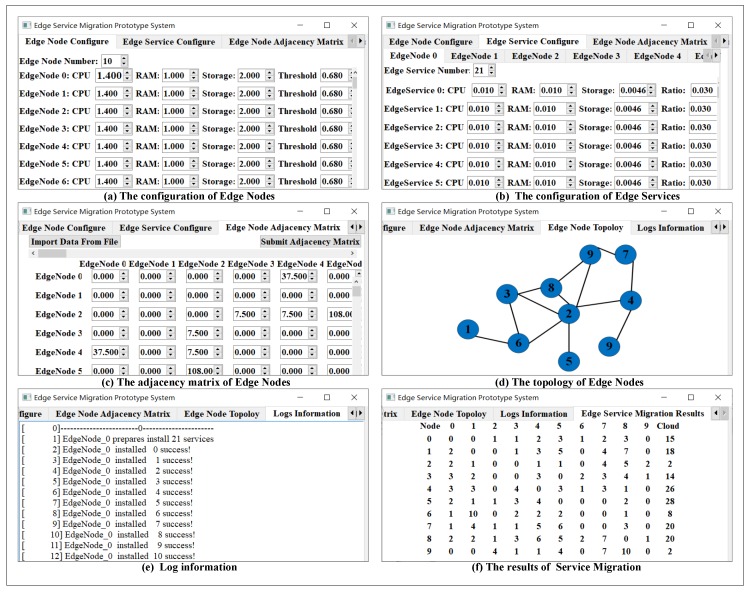
The user interface of the prototype system.

Figure 4 shows the Configuration of the prototype system. The simulated information of edge nodes needs to be configured in the sub-tab of the *EDGE NODE CONFIGURE UI*, including the number of edge nodes, CPU, RAM, and Storage. And the value of the threshold $-\theta_{max}$ is configured as shown in Figure 4a. After setting the edge nodes, the services of each edge nodes need to be set in the sub-tab of *EDGE SERVICE CONFIGURE UI*, including the number and resource requirement of edge servicesas shown in Figure 4b. The bandwidth between edge nodes is configured in the sub-tab of *EDGE NODE ADJACENCY MATRIX UI*, the configure information can be imported from a file as shown in Figure 4c. After submitting the configure information of bandwidth, the topology is got from the sub-tab of *EDGE NODE TOPOLOGY UI* as shown in Figure 4d. Finally, the system can be started to simulate the service migration and show the real-time information in LOGS INFORMATION UI as shown in Figure 4e. When this simulation system finishes the work of smart service migration, the total number of service migration among edge nodes is showed in the sub-tab of *EDGE SERVICE MIGRATION RESULT UI* as shown in Figure 4f.

### 4.3. A Case of Edge Service Migration on Prototype System

In this section, a case of edge service migration is simulated based on the prototype system. In this case, it has 10 edge nodes and a cloud center. Figure 4d shows the topology of the 10 edge nodes. Table 2 is the size of bandwidth between edge nodes.

The allocating resource of edge nodes depends on the hardware of Raspberry pi 3. For example, edge nodes include three types of internet that are 7.5 MB/s Bluetooth, 37.5 MB/s Wi-Fi and the 108.0 MB/s wired. In addition, the size of CPU, RAM, and storage are 1.4 GHz, 1 GB and 2 GB, respectively. Finally, the storage size of edge services is referred to some smart services from the domain of deep learning as shown in Table 3.

**Figure 5:**
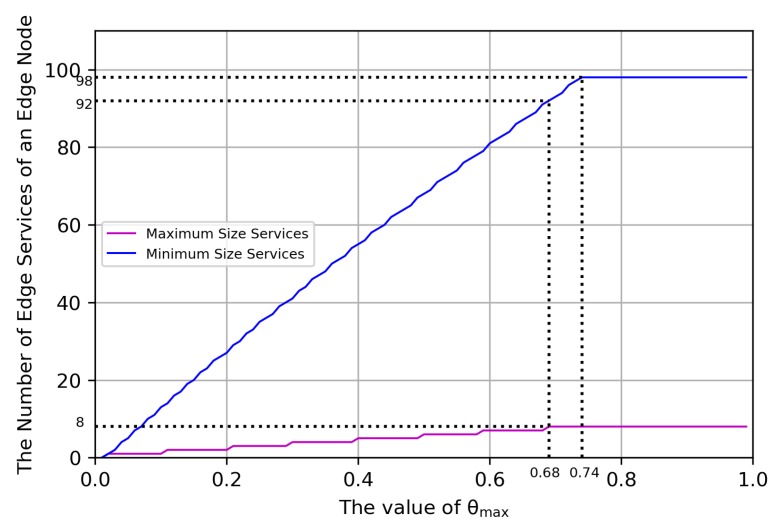
The relationship between the value of $\theta_{max}$ and the total number of edge services.

The value of $\theta_{max}$ is a critical parameter and impacts the performance of the edge network. In order to get an optimal value, two group experiments are done. One is that all the edge smart services are the maximum size of smart services, that is, 548.051 MB. The other is that all the edge smart services are the minimum size of smart services, that is, 4.736 MB. The result is shown in Figure 5. The value of $\theta_{max}$ needs to satisfy $0.68\leq \theta_{max}\leq 0.74$. In order to make the edge network adapt to the extreme conditions, all the edge services are in the maximum size. In this way, the value of $\theta_{max}$ had better be set to 0.68.

In the simulation experiment, the number of services in each edge node is randomly generated, and the type of each edge service is randomly selected from Table 3. The experiment result of service migration is shown in Table 4.

According to the experiment result, the total number of hosting services is 348 and the total number of migrating services among edge nodes is 195. In addition, the total migration storage size is 92,1963 GB. Moreover, the total execution time of service migration among edge nodes is 241 s. Compared with the cloud migration, the efficiency of edge service migration is improved about three times.

## 5. Conclusions

This paper has proposed a resource-constrained smart service migration framework for the edge computing environment in IoT and a dynamic edge service migration algorithm. The IoT-RECSM can ensure the resource load of IoT edges smoothly, by migrating some services of resource-critical nodes to resource-rich nodes. A smart service migration method has also constructed for the IoT-RECSM, which can be deployed on the IoT edge to dynamically monitor and migrate services. Finally, a smart service migration prototype system has been implemented to simulate the service migration based on IoT-RECSM. An IoT case including 10 nodes is simulated to evaluate our approach. According to the experiment results, service migration among edge nodes not only maintains the stability of service execution on edge nodes, but also reduces the sensor data traffic between edge nodes and cloud center.

There are still some limitations to be addressed and ongoing work for the IoT-RECSM. Currently, the simulation of migration delay in the algorithm is not universal, cannot deal with the Pre-copy migration and Post-copy migration very well. We will improve the simulation method of migration delay to adapt to live migration in future work. Moreover, the dynamical service migration approach of IoT-RECSM is complex. It needs to consume some additional resources to compute the parameters of migration models in real-time, which may restrict the efficiency of service migration. We intend to adopt the learning technologies into the edge service migration. It pre-learns a migration model for every node according to the historical log information of resource consumption. And then, a distributed migration algorithm can be easily designed for the nodes.

## Figures and Tables

**Table 1:** Symbol descriptions for basic elements.

Symbol	Description
*V*	The set of edge nodes and ‖V‖ is the number of edge nodes.
$v_{i},v_{t}$	Edge node *i* and *t* and $v_{i},v_{t}\in V$.
$S_{v_{i}}$	The set of edge services of edge node $v_{i}$.
$\|S_{v_{i}}\|$	The number of edge services of edge node $v_{i}$.
*S*	The set of edge services.
$S_{v_{i},j}$	The j-th service of edge node $v_{i}$.
TR	The set of total resource of edge nodes.
$TR_{v_{i},k}$	The total resource of k-th type of $v_{i}$.
$r_{v_{i}}$	The number of resource types of edge node $v_{i}$.
TCR	The set of total cost resource
$TCR_{v_{i}}$	The set of total cost resource of $v_{i}$ edge nodes.
$TCR_{v_{i},k}$	The cost resource of k-th type of $v_{i}$.
SCR	The set of total cost resource of edge services.
$SCR_{s_{v_{i}},j,k}$	The cost resource of k-th type of $v_{i}$’s j-th service.
exp(.)	The exponential function.
$size(S_{v_{i},t})$	The storage size of t-th service of edge node $v_{i}$.
${℘}_{v_{i}}$	The value of resource utilization of $v_{i}$.
$l_{S_{v_{i},j}}$	The value of resource usage of $v_{i}$’s j-th service.

**Table 2:** The bandwidth between edge nodes.

Edge Node	0	1	2	3	4	5	6	7	8	9
0	0.0	0.0	0.0	0.0	37.5	0.0	0.0	0.0	0.0	0.0
1	0.0	0.0	0.0	0.0	0.0	0.0	37.5	0.0	0.0	0.0
2	0.0	0.0	0.0	7.5	7.5	108.0	7.5	0.0	108.0	37.5
3	0.0	0.0	7.5	0.0	0.0	0.0	7.5	0.0	7.5	0.0
4	37.5	0.0	7.5	0.0	0.0	0.0	0.0	37.5	0.0	0.0
5	0.0	0.0	108.0	0.0	0.0	0.0	0.0	0.0	0.0	0.0
6	0.0	37.5	7.5	7.5	0.0	0.0	0.0	0.0	0.0	0.0
7	0.0	0.0	0.0	0.0	37.5	0.0	0.0	0.0	0.0	108.0
8	0.0	0.0	108.0	7.5	0.0	0.0	0.0	0.0	0.0	108.0
9	0.0	0.0	37.5	0.0	0.0	0.0	0.0	108.0	108.0	0.0

**Table 3:** Service and its size.

Service	Storage Size (MB)	Service	Storage Size (MB)
squeezenet1_1 [31]	4.736	resnet101 [32]	170.449
squeezenet1_0 [31]	4.785	resnet152 [32]	230.341
densenet121 [33]	30.844	alexnet [34]	233.095
resnet18 [32]	44.658	vgg11 [35]	506.835
densenet169 [33]	54.708	vgg11_bn [35]	506.881
densenet201 [33]	77.373	vgg13 [36]	507.540
resnet34 [32]	83.261	vgg13_bn [36]	507.589
resnet50 [37]	97.753	vgg16 [35]	527.795
googlenet [38]	103.814	vgg19 [36]	548.051

**Table 4:** The number of migrating services in every edge node and cloud.

Edge Node	0	1	2	3	4	5	6	7	8	9	Cloud
0	0	0	1	1	2	3	1	2	3	0	15
1	2	0	0	1	3	5	0	4	7	0	18
2	2	1	0	0	1	1	0	4	5	2	2
3	3	2	0	0	3	0	2	3	4	1	14
4	3	3	0	4	0	3	1	3	1	0	26
5	2	1	1	3	4	0	0	0	2	0	28
6	1	10	0	2	2	2	0	0	1	0	8
7	1	4	1	1	5	6	0	0	3	0	20
8	2	2	1	3	6	5	2	7	0	1	20
9	0	0	4	1	1	4	0	7	10	0	2
